# Emerging Ferroptosis Involvement in Amyotrophic Lateral Sclerosis Pathogenesis: Neuroprotective Activity of Polyphenols

**DOI:** 10.3390/molecules30061211

**Published:** 2025-03-08

**Authors:** Annamaria Russo, Stefano Putaggio, Ester Tellone, Antonella Calderaro, Santa Cirmi, Giuseppina Laganà, Silvana Ficarra, Davide Barreca, Giuseppe Tancredi Patanè

**Affiliations:** Department of Chemical, Biological, Pharmaceutical and Environmental Sciences, University of Messina, 98166 Messina, Italy; arusso@unime.it (A.R.); anto.calderaro@gmail.com (A.C.); santa.cirmi@unime.it (S.C.); giuseppina.lagana@unime.it (G.L.); sficarra@unime.it (S.F.); davide.barreca@unime.it (D.B.); giuseppe.patane@studenti.unime.it (G.T.P.)

**Keywords:** natural compounds, oxidative stress, neurodegenerative diseases, system Xc^−^, cell death, glutathione peroxidase 4

## Abstract

Neurodegenerative diseases are a group of diseases that share common features, such as the generation of misfolded protein deposits and increased oxidative stress. Among them, amyotrophic lateral sclerosis (ALS), whose pathogenesis is still not entirely clear, is a complex neurodegenerative disease linked both to gene mutations affecting different proteins, such as superoxide dismutase 1, Tar DNA binding protein 43, Chromosome 9 open frame 72, and Fused in Sarcoma, and to altered iron homeostasis, mitochondrial dysfunction, oxidative stress, and impaired glutamate metabolism. The purpose of this review is to highlight the molecular targets common to ALS and ferroptosis. Indeed, many pathways implicated in the disease are hallmarks of ferroptosis, a recently discovered type of iron-dependent programmed cell death characterized by increased reactive oxygen species (ROS) and lipid peroxidation. Iron accumulation results in mitochondrial dysfunction and increased levels of ROS, lipid peroxidation, and ferroptosis triggers; in addition, the inhibition of the Xc^−^ system results in reduced cystine levels and glutamate accumulation, leading to excitotoxicity and the inhibition of GPx4 synthesis. These results highlight the potential involvement of ferroptosis in ALS, providing new molecular and biochemical targets that could be exploited in the treatment of the disease using polyphenols.

## 1. Introduction

Amyotrophic lateral sclerosis (ALS) is a progressive and ultimately fatal neurodegenerative disease characterized by growing muscle paralysis caused by the degeneration of motor neurons in the primary motor cortex, brainstem, and spinal cord, typically leading to mortality within 2–5 years of symptom onset [1]. The disease mainly affects men and the elderly, aged between 65 and 85 years, while it rarely affects children [2,3,4]. Generally, neurological disorders tend to selectively affect one type of motor neuron: the upper motor neurons that originate in the motor cortex of the brain and carry electrical impulses to the lower motor neurons that originate in the spinal cord and transmit the impulses received to the skeletal muscles. ALS, unfortunately, affects both. The degeneration of motor neurons causes the progressive loss of their ability to formulate and transmit impulses, and the consequent loss of motor control. As the disease and neuronal degeneration progress, there is an exacerbation of the symptoms; this start with general muscle weakness and then results in the patient being forced to use a wheelchair due to a total loss of motor control. Death generally occurs due to respiratory failure caused by the degeneration of the motor neurons that innervate the diaphragm [5]. Typical symptoms of ALS include slow and progressive muscle weakness, cramps, muscle atrophy and stiffness, dysphagia, difficulty speaking and swallowing, and weakening of the respiratory muscles, which can lead to respiratory failure and death; moreover, as the disease progresses, difficulties in the use of the limbs and trunk may be experienced, as well as problems with posture and gait control [6,7,8]. Although several studies have been undertaken, the causes of the disease are still unknown, and there are no specific tests for the diagnosis of ALS, which is made only through the evaluation of symptoms and clinical signs. In addition, from a pharmacological point of view, there are currently only three drugs approved for ALS: riluzole, edaravone, and AMX0035. These slow down the progression of symptoms, but the effect is short term [9,10,11]. The pathological hallmarks of ALS include alterations in the homeostasis of iron, and more generally of metals, oxidative stress, mitochondrial dysfunction, and alterations in glutamate metabolism. Peculiarly, these are all also characteristics of a biochemical process of programmed cell death known as ferroptosis [12,13,14,15]. In this context, the aim of this review is to investigate the potential contribution of ferroptosis in the pathogenesis of ALS. Ferroptosis is a new form of cell death linked to iron metabolism, reactive oxygen species (ROS) accumulation, and lipid peroxidation that was first discovered by Stockwell and described by Dixon in 2012 [16,17]. The process is implicated in the development of many diseases, such as cancer, stroke, myocardial infarction, and neurodegenerative diseases, including Alzheimer’s disease (AD), Huntington’s disease (HD), Parkinson’s disease (PD), and ALS [15]. A comprehensive understanding of the implications of ferroptosis in the progression of ALS pathogenesis may offer new insights into novel therapies aimed at enhancing the clinical outcomes of patients. Furthermore, understanding the pathological convergence between these neurodegenerative disorders will help open new avenues for early diagnosis and the development of innovative treatments that target shared mechanisms.

## 2. Pathophysiology of ALS

**Figure 1:**
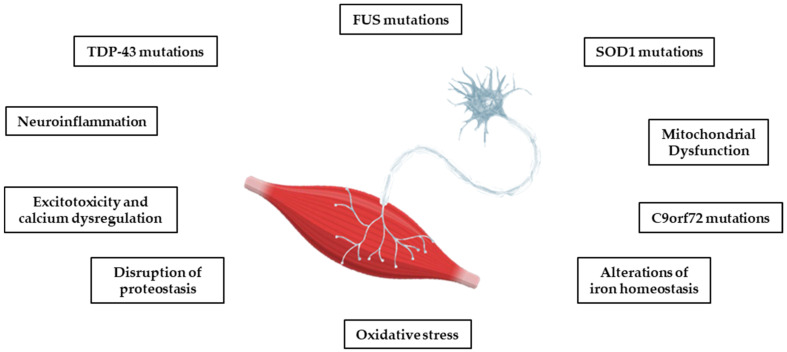
The main pathophysiological processes involved in ALS. The pathophysiological processes of the disease are complex, and several mechanisms contribute to neurodegeneration, with multiple sequential steps. The onset of pathology appears to be due to a heterogenous interaction of genetic, epigenetic, and environmental factors.

Most cases of ALS are sporadic (sALS), but in 10–15%, there is a clear family history (fALS) and a cause can be detected in the mutations of several genes, including superoxide dismutase 1 (SOD1), Tar DNA binding protein 43 (TDP-43), Chromosome 9 open frame 72 (C9ORF72), and Fused in Sarcoma (FUS) [18]. These are rare mutations that today tend to be considered important even in the absence of a family history [19]. However, the risk of developing ALS and the factors controlling the onset and progression of the disease seem to be genetically independent, although all the mutations studied converge on targeted biological pathways. Some of these pathways, including oxidative stress, the dysregulation of mitochondrial function, protein homeostasis, glutamate toxicity, and calcium dysregulation, also play a key role in other neurodegenerative diseases, such as AD, PD, and HD (see Figure 1) [12,13]. Although the molecular pathway responsible for motor neuron degeneration in ALS is not yet known, there is evidence for a complex interaction of several pathogenic cellular processes that may not be mutually exclusive.

### 2.1. Gene Mutations

Several genetic mutations have been associated with the disease, but only a few of them are linked to a significant percentage of ALS cases, such as SOD1, TARDBP, C9ORF72, and FUS genes [20]. Gene mutations may cause the synthesis of defective proteins that could damage the cell and lead to the disease. A mutation in the SOD1 gene, one of the key contributors to ALS, results in misfolding and the aggregation of the protein copper/zinc-binding superoxide dismutase 1 (SOD1). The enzyme catalyzes the dismutation of superoxide radicals ($O_{2}^{.-}$) into hydrogen peroxide (H_2_O_2_) and molecular oxygen (O_2_) [21]. Mutant SOD1 in ALS triggers misfolding and aggregation via abnormal disulfide cross-linking, forming toxic inclusions in the mitochondrial intermembrane space of neuronal cells, causing mitochondrial dysfunction and oxidative stress, two crucial events for disease progression [22,23]. Rare mutations in TARDBP, the gene encoding TDP-43—a critical DNA/RNA binding protein—cause its hyperphosphorylation and protein mislocalization. TDP-43 is mainly located in the nucleus and is involved in mRNA stabilization and transcriptional regulation. Under pathological conditions, post-translational modifications of TDP-43 cause the protein’s escape from the nucleus, abnormal aggregation, and cytoplasmic accumulation [24,25,26]. Renton et al. demonstrated that a pathogenic hexanucleotide expansion in the C9ORF72 gene is a major genetic cause of ALS and frontotemporal dementia (FTD), in association with chromosome 9p21 [27]. Repeated expansion causes neuronal toxicity through the aggregation of repeated RNA and dipeptide protein products [28]. Finally, FUS mutations represent the third most significant cause of fALS in Italy, after SOD1 and TARDBP [29]. The FUS gene encodes a ubiquitously expressed DNA/RNA-binding protein mainly confined in the nucleus, whereas in ALS motor neurons it is localized in the cytoplasm. This mislocalization of mutant FUS leads to a cascade of damaging events that lead to neurodegeneration [30]. The FUS protein consists of three structural domains: an N-terminal region that serves as the transcriptional activation domain, an RNA-binding domain, and a C-terminal domain, which contains the nuclear localization signal [29]. Mutations in FUS can alter the physiological functions of the protein, leading to altered synaptic activity, mitochondrial dysfunction, oxidative stress, and ultimately neuronal damage and death.

### 2.2. Excitotoxicity and Calcium Dysregulation

Excitotoxicity is a pathological condition related to the overstimulation of α-amino-3-hydroxy-5-methyl-4-isoxazole propionic acid (AMPA) receptors, which occurs when there is an excitatory response due to the prolonged release of glutamate, the main excitatory neurotransmitter of the central nervous system (CNS), which leads to an increase in the intracellular influx of calcium ions (Ca^2+^). The increase in ions in the cellular environment, on the one hand, leads to the depolarization of the mitochondrial membrane inhibiting the respiratory chain, and on the other hand determines the overactivation of ATP-dependent ion pumps, which stabilizes calcium homeostasis, reducing the intracellular concentration of ATP. This stress condition is increased by the activation of catalytic enzymes and the production of ROS and reactive nitrogen species (RNS), leading to further cellular damage [31]. Several studies have also shown that motor neurons are remarkably sensitive to this process and to oxidative stress due to their size; in fact, these cells have a diameter of about 100 μm, and an axon can extend up to one meter away, meaning that a considerable amount of oxygen and energy is required to perform their functions. In addition, the spinal motor neurons express a high number of Ca^2+^-permeable AMPA receptors, which determine the internalization of ions, and a relatively low concentration of ion transporter proteins, which can facilitate Ca^2+^ release from intracellular stores. This unique characteristic of motor neurons is one of the major causes of their vulnerability to excitotoxicity [32]. The link between ALS and altered glutamate transmission is potentially explained through the involvement of astrocytes; in fact, these cells, under normal conditions, are equipped with an excitatory amino acid transporter system 2 (EAAT2) for glutamate; in pathological conditions, on the other hand, the presence of EAAT2 is lower, and this does not allow the reabsorption of the neurotransmitter. In addition, Sasaki et al. observed in the spinal cord of ALS patients an increase in the cysteine/glutamate antiport system (system Xc^−^), which on the one hand restabilizes the production of reduced glutathione (GSH), counteracting oxidative stress, and on the other hand increases glutamate intake by promoting excitotoxicity [33]. This condition causes an imbalance in the concentration of transition metals that leads to an alteration in the cytoplasm [Ca^2+^], contributing to impaired motor neuron function [34].

### 2.3. Neuroinflammation

Neuroinflammation is a process that occurs when glial cells or circulating cells of the immune system interact with cells in the CNS during infection, degeneration, or injury; if the damage that triggered the inflammatory response is not repaired and persists over time, this can lead to complications and an aggravation of the pathological condition. Postmortem screening in individuals with ALS revealed the abnormal proliferation and activation of glial cells and the presence of T cells in disease-affected regions, showing the involvement of these cells in disease progression [35,36]. Among the glial cells, microglia cells, highly specialized macrophages in the CNS, are the most involved. These cells may have an inflammatory (M1) or anti-inflammatory (M2) phenotype: M1 is a toxic phenotype that can lead to the production of ROS and proinflammatory cytokines, while M2 is a protective phenotype that produces neurotrophic factors and anti-inflammatory cytokines [37].

Microglia activation is a feature of ALS; its reactivity has been specifically detected in the regions affected by the disease, supporting the idea that microglia may contribute to the progression of neurodegeneration [38]. Liao et al., in a study on adult mice with ALS (mSOD1^G93A^), observed that during the presymptomatic stages of the disease, there was an overexpression of microglia with the M2 phenotype, and as the disease progressed there was a conversion to the M1 phenotype that stimulated the production of prooxidant enzymes, such as nitric oxide synthase (NOX), inducible nitric oxide synthase (iNOs), and cytokine release [39]. On the other hand, Rossi et al. observed that the use of interleukin 4 (IL-4) in mSOD1^G93A^ mice can modulate the differentiation of microglia cells towards the M2 phenotype, improving the course of ALS pathology [40]. In addition to microglial cells, astrocytes, the main glial cells of the CNS, are also involved in ALS. Some in vivo studies have shown a neurotoxic phenotype of these cells that causes damage to motor neurons. This induced damage can be direct or indirect: in the first case, the damage is linked to the release of pro-inflammatory cytokines and ROS; in the second case, it is linked to the interaction of the cells with the immune system T cells [41]. As mentioned above, T lymphocytes, which include CD4+ T and CD8+ T cells, are also involved in the inflammatory response of the CNS and in the progression of neurodegeneration [42]. In fact, it has been seen that in the early stages of the disease, CD4+ T cells promote the secretion of IL-4, which positively modulates microglial cells, inducing the M2 phenotype; as the disease progresses, however, these cells acquire an inflammatory Th1/Th17 phenotype, resulting in the release of inflammatory cytokines, ROS generation, and microglia conversion from M2 to M1 [43]. CD8+, on the other hand, has a double action: it promotes myelin regeneration at the level of the peripheral nervous system and has neurotoxic action at the level of the spinal cord [44,45].

### 2.4. Mitochondrial Dysfunction

Mitochondria play important roles within cells producing energy in the form of ATP, mediating lipid metabolism, apoptotic processes, and calcium homeostasis [46,47]. In neurons, the efficiency of these organelles is also important for axonal development and regeneration, as well as for synaptic function [48]. Failure and/or impaired functioning of the mitochondria at the neuronal level leads to cell damage and neurodegenerative disorders, including ALS. Mitochondrial dysfunction in ALS can present in different forms, such as alterations in oxidative phosphorylation, manifested by the reduced function of complex I and complex II of the respiratory chain, the production of ROS, etc.; moreover, from a morphological point of view, the neurons of subjects with ALS have mitochondria with a swollen and vacuolated appearance [49,50]. It has been shown that SOD1, TDP-43, FUS, C9ORF72, and the dipeptide repeater protein glycine/arginine (GR), associated with the expansion of the GGGGCC repeat (DPR) C9ORF72, interfere with mitochondria, causing their alteration in cases of ALS [51]. Specifically, the aggregation of mutated SOD1 at the level of the mitochondrial intermembrane space reduces the activity of the electron transport chain; in addition, the protein aggregates, resulting in the inhibition of voltage-gated anion channel 1 (VDAC1) activity, ADP permeability, and increased ALS progression [52,53]. TDP-43 and the ALS mutant TDP-43 accumulate in the mitochondrial cytosol, resulting in the breakdown of complex I of the electron transport chain [54]. Also, FUS protein accumulation in the mitochondria causes ATP production to decrease and increased ROS generation [55].

### 2.5. Oxidative Stress

Several pieces of experimental evidence indicate a key role for oxidative stress in the pathophysiology of ALS [56,57,58]. Oxidative stress is a peculiar process characterized by the imbalance between the generation of ROS and the antioxidant capacity of the cells found in several pathological conditions. ROS accumulation and the trigger of oxidative stress may contribute to exacerbating many pathological processes involved in motor neuron damage, and are known to be the predominant causes of cellular death [59,60,61]. This is one of the reasons that drugs with oxidative stress as a specific target could be the most promising therapeutic candidates for the prevention of motor neuron degeneration [62]. In subjects with ALS, elevated levels of ROS have been found in different areas of the CNS, showing redox homeostasis compromission in the progression of the disease [51,63,64,65,66]. The accumulation of ROS could be linked to the mutation of SOD1 [67]. Misfolded SOD1 binds to optineurin, an autophagy receptor; the binding impairs mitophagosome formation, leading to the accumulation of ROS and dysfunctional mitochondria [68,69]. Damage to the mitochondria causes an alteration in cytosolic calcium levels that increases ROS generation and directs the cell towards apoptosis; in addition, Weiduschat et al. showed that GSH concentration, at the level of the motor cortex, is reduced in subjects with ALS compared to healthy subjects by about 31% [70]. Meanwhile, in studies conducted by Killoy et al. and Pehar et al. on murine models, it has been observed that the decrease in GSH causes an increase in the speed of motor neuron degeneration [71,72]. In ALS subjects, the detection of biomarkers for oxidative stress at the level of cerebrospinal fluid, tissue, blood, and urine revealed an increase in carbonylated and/or glycosylated proteins, lipid peroxidation, and DNA damage [73].

### 2.6. Alterations in Metal Ion Homeostasis

Metal ions, such as iron (Fe), copper (Cu), and zinc (Zn), are widely distributed in the brain and play a central role in CNS function. Fe, for example, is involved in myelin synthesis, oxidative phosphorylation, neurotransmitter production, etc.; Cu, on the other hand, in addition to being involved (together with Zn) in the type 1 antioxidant enzyme SOD1, is the cofactor of cytochrome c oxidase and contributes to neuronal excitability [74,75]. Copper plays a fundamental role in the CNS because it acts as a cofactor for multiple oxidoreductase enzymes, intervenes in electron transport and neurotransmitter synthesis, participates in myelination, and helps to regulate iron homeostasis (oxidizing Fe^2+^ into Fe^3+^ through binding to ceruloplasmin) [76,77]. Some studies have highlighted altered copper distribution, decreased ferroxidase activity, and the generation of a pool of SOD1 that lacks Cu in the catalytic site in human sporadic ALS [78]. The key role of copper is also supported by the therapeutic mechanism of action for diacetylbis(N(4)-methylthiosemicarbazonato) copper(II) (CuII(atsm)), one of the most tested drugs developed for the treatment of ALS [79,80].

Iron, on the other hand, is internalized in the brain in its ferric form (Fe^3+^) and is bound to transferrin through the transferrin receptor membrane protein 1 (TfR1) located in the blood–brain barrier (BBB) [81]. It is then released by transferrin, reduced to a ferrous ion (Fe^2+^) by the enzyme ferrireductase, and absorbed by brain transferrin, before being synthesized by oligodendrocytes (they store most of the iron in the CNS) [82,83]. At this point, iron can be reoxidized to Fe^3+^ through the Fenton reaction, or excess iron is accumulated by cytosolic ferritin [84]. Several studies have highlighted iron as a marker in patients with ALS; the progression of the disease is associated with changes in proteins involved in iron metabolism and increased skeletal muscle iron accumulation [85,86,87]. The biochemical pathway of this dyshomeostasis is mediated by the inactivation of protein kinase B (Akt), which is responsible for the upregulation of iron import and storage proteins such as ferritin, but not proteins involved in iron export, such as ferroportin [88]. Excess iron accumulation and its redox activity can lead to the generation of ROS and RNS, resulting in membrane peroxidation and cell damage. This iron-dependent oxidative stress condition in ALS negatively influences signaling pathways and may be associated with a novel mechanism of programmed cell death known as ferroptosis. In this context, initial studies have suggested a significant involvement of ferroptosis in ALS progression [89,90]. Several cellular and molecular processes reported as markers of ferroptosis are also found in ALS, including oxidative stress and lipid peroxidation, mitochondrial dysfunction, glutamatergic excitotoxicity, and deficiencies in glutathione peroxidase 4 (GPx4) [89,91,92,93].

## 3. Ferroptosis

**Figure 2:**
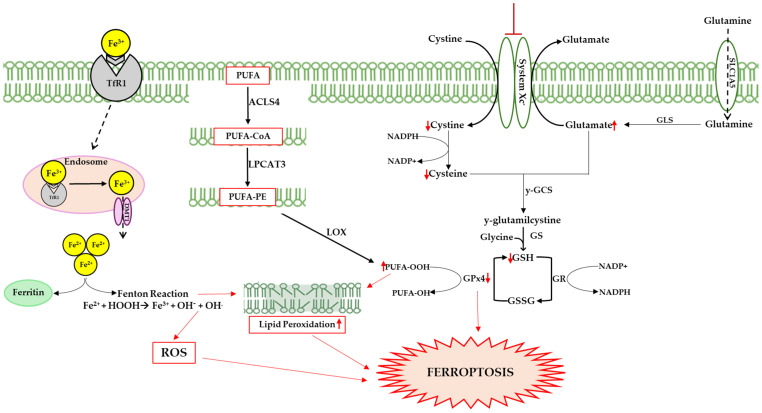
Ferroptosis can be triggered by several pathways; one of these involves transferrin, following the release of iron from the internalized protein after its binding to transferrin receptor 1 (TfR1). Cytosolic iron accumulation causes excessive ROS generation via the Fenton reaction (Fe^2+^ + H_2_O_2_ → Fe^3+^ + ·OH + OH^−^), altering the delicate balance between the physiological and pathophysiological roles of ROS. Highly reactive hydroxyl radicals (·OH) interact with polyunsaturated lipids, triggering lipid peroxidation and damaging membrane integrity. Hydroperoxide radical production is also linked to the action of lipoxygenases (LOXs), which catalyze stereospecific oxygen addition to long chain fatty acids (PUFAs). Specifically, acyl-coenzyme A synthetase long-chain family member 4 (ACSL4) catalyzes the addition of Coenzyme A to fatty acids to produce PUFA-CoAs, which are utilized by lysophosphatidylcholine acyltransferase 3 (LPCAT3) to produce PUFA-PE, promoting lipid peroxidation. Another pathway could involve the inhibition of system Xc^−^, a membrane transporter that allows the exchange of cystine and glutamate in and out of the cell. The inhibition of the transporter causes a reduction in cystine in the cellular environment that results in GSH synthesis, and consequently reduced GPx4 efficiency. This enzyme prevents lipid peroxidation, neutralizing hydroperoxides through GSH oxidation. Up and down red arrows indicate the increase and decrease of concentration, respectively.

From a biological point of view, ferroptosis is characterized by alterations in iron homeostasis, the accumulation of Fe^2+^, the reduced activity of GPx4, reductions in GSH, and increases in ROS and lipid peroxidation (see Figure 2). In addition, morphological changes in the mitochondria are evident, with a narrowing of their size, reductions in the mitochondrial crests, thickening of the membrane, and rupture of the outer membrane [94].

### 3.1. Iron Metabolism

Iron accumulation in the brain is an emblematic finding of aging and neurodegeneration [95,96,97]. The brain acquires iron through transferrin receptor protein 1 (TfR1)-mediated endocytosis via TfR1/divalent metal transporter 1 (DMT1); the ions are then used for neuronal and glial metabolism for the electron transport biosynthesis of metalloproteins or neurotransmitters [98]. If in excess, iron is stored in ferritin as Fe^3+^ or exported into the circulation via ferroportin1, with oxidation of the extracellular metal via ceruloplasmin or hephestin [99,100]. Numerous studies have shown that ferritin concentration increases with age, promoting iron overload and contributing to the decline in cognitive function [101,102,103]. Halon-Golabek et al. demonstrated that ALS is associated with the impairment of iron metabolism. The progression of the disease induces the downregulation of AKT, which causes the upregulation of proteins involved in iron import and storage (ferritin L, H, TfR1), while the proteins involved in iron export (ferroportin) decrease [80]. Iron overload is the main cause of ROS production, leading to oxidative stress, lipid peroxidation, and ferroptosis [104].

### 3.2. Lipid Peroxidation

Ferroptosis differs from other forms of cell death by its high levels of lipid peroxidation, a peculiar process in which oxidizing agents such as ROS target carbon–carbon double bonds in membrane phospholipids, particularly PUFAs [105,106]. Lipid peroxidation can be triggered either through a non-enzymatic spontaneous autoxidation mechanism or through an enzymatic mechanism (see Figure 2). In the first case, the excess of free iron in the cellular environment can lead to the formation of radicals through the Fenton reaction, triggering the ferroptotic process. In the second case, PUFAs are oxidized by LOXs, forming hydroperoxide derivatives; these are very reactive compounds that lead to the production of aldehydes such as 4-hydroxynonenal and malondialdehyde, and which trigger a cascade of chain reactions on the membrane, compromising its integrity [107,108,109].

### 3.3. Antioxidant Systems Involved in Ferroptosis

The body is characterized by a wide range of antioxidant agents; among these, GSH is the most important endogenous antioxidant that acts as a substrate of GPxs [110]. GPxs are a family of enzymes that catalyze reductions in hydroperoxides, including lipid peroxides (LOOH) in H_2_O and the corresponding alcohols via the oxidation of GSH in GSSG (disulfide form). Among the isoforms, GPx4 is closely associated with ferroptosis and plays an important role as an antagonist of the process by clearing intracellular peroxides and maintaining cell survival [111,112]. The catalytic activity of GPx4 is crucially modulated by GSH intracellular concentrations, which in turn are tightly regulated by the activity of the Xc^−^ system, an antiporter of cystine/glutamate composed of two chains, the light chain SLC7A11 (Solute Carrier Family 7 Member 11) and the heavy chain SLC3A2 (Solute Carrier Family 3 Member 2). GSH is synthesized in the cytosol from glutamate, glycine, and cysteine, with the latter being the rate-limiting factor for tripeptide synthesis. Cysteine uptake in the cell due to the Xc^−^ system is a speed-limiting factor for GSH biosynthesis [110,113,114,115]. In summary, the inhibition of the Xc^−^ system causes a reduction in the entry of cystine into the cellular compartment, resulting in the inhibition of GSH synthesis; this causes a reduction in the activity of GPx4 and consequently an alteration in the redox homeostasis of the cell, a lethal accumulation of ROS, and the start of the ferroptotic process [112]. A reduced expression of GPx4, the growth of phospholipid peroxides, and a resulting loss of motor neurons have been shown in the spinal cords of ALS mice [93].

### 3.4. Erythroid Nuclear Factor 2-Related Factor

Nrf2 is an endogenous antioxidant transcriptional activator belonging to the cap‘n’collar-basic region leucine zipper (CNC-bZIP) family; it is capable of regulating cell-cycle homeostasis and cell protection through interactions with antioxidant response elements (AREs) [116,117]. Under physiological conditions, Nrf2 is bound to Kelchlike ECH-associated protein 1 (Keap1) in the cytoplasm; in the case of oxidative stress, these detach and translocate to the nuclear level, forming heterodimers with small musculoaponeurotic fibrosarcoma (sMAF) proteins. This allows interaction with ARIS and the regulation of the expression of antioxidant enzymes. Structurally, Nrf2 consists of seven domains ranging from Neh1 to Neh7. In detail, Neh1 is made in the CNC-bZIP region, where DNA binds sMAF proteins as dimerization partners of Nrf2; Neh2 is the main negative regulation site of Nrf2, consisting of two domains, DLG and ETGE. The latter shows high affinity with Keap1; Neh3 has the ability to regulate the transcription of genes in the ARE region. Neh4 and Neh5 are acidic amino-acid-rich regions capable of interacting with the binding protein of the cAMP co-activator response element, initiating the transcriptional process of the binding of the Nrf2-MAF dimer to the AREs. Neh6 is a serine-rich domain that regulates the degradation of Nrf2; Neh7 inhibits Nrf2 transcription [118,119,120,121,122]. Nrf2 activity reduces ROS production at the mitochondrial level, influences organelle biogenesis, promotes GSH biosynthesis and NADPH restoration (regulates GPx4 function), and regulates lipid and iron metabolism [123,124,125,126].

## 4. Ferroptosis and ALS

Neurodegenerative diseases, such as AD, PD, HD, and ALS, as the leading causes of disability worldwide, are frequently characterized by overlapping aspects of pathology beyond neuronal loss. Several studies have suggested common pathological pathways between neurodegenerative diseases regarding the aggregation of toxic proteins, leading to the loss of neuronal function and triggering progressive cell death [127,128,129,130,131]. These conditions can be associated with a local inflammatory state due to iron accumulation, which, together with high oxygen consumption and a great concentration of PUFAs (both peculiar characteristics of the brain), causes an increase in oxidative stress and makes neurons particularly vulnerable to the triggering of ferroptotic damage [101,132,133]. Specifically, decreases in antioxidant systems, including GPxs and alterations in iron homeostasis, are closely related to AD progression and cognitive decline. Patients with AD showed an increase in iron due to the upregulation of ferritin and downregulation of ferroportin in the brain, increasing iron intake and reducing iron excretion, respectively [134,135]. In addition, Bao et al., after the injection of Aβ into the brains of mice, observed decreased levels of GPx4, suggesting a direct pathological involvement of Aβ in neuronal ferroptosis [136]. Ferroptosis features are common also of PD patients, including GSH depletion, increases in ROS generation, and lipid peroxidation [137,138]. In addition, in Parkinsonism, iron economy of the extrapyramidal system is abnormal, and alterations in iron deposition are negatively correlated with the severity of PD [139]. Iron accumulation leads to the death of dopaminergic neurons through various pathways, including mitochondrial dysfunction [140,141]. High levels of lipid peroxidation are the main feature of HD. Numerous studies have demonstrated significantly low GSH levels in the plasma associated with decreased GPx4 activity [142,143]. The content of ferritin in the striatum is also significantly increased, and treatment with ferroptosis inhibitors and iron chelators significantly reduced the death of nerve cells in the brains of HD rats [144,145].

**Figure 3:**
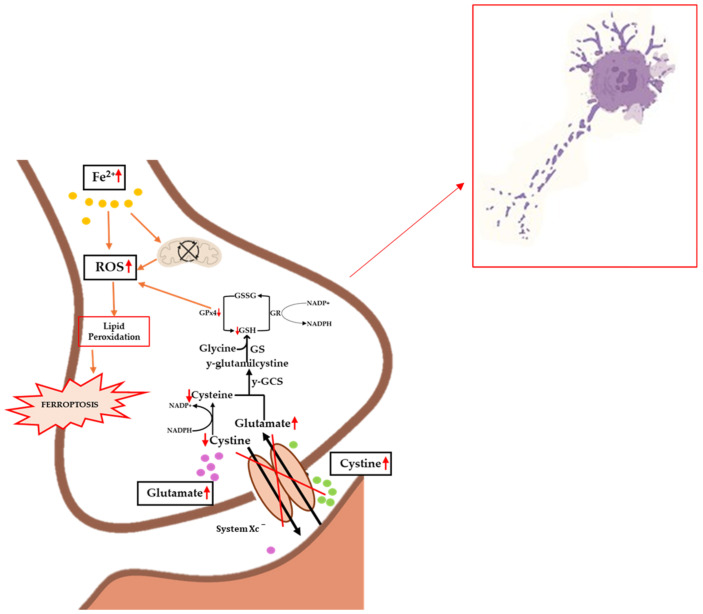
Implications of ferroptosis in the biochemical progression of ALS. Iron accumulation causes mitochondrial dysfunction and increased levels of ROS, lipid peroxidation, and ferroptosis. Inhibition of the Xc^−^ system causes decreased levels of cystine and the accumulation of glutamate, which on the one hand results in excitotoxicity and on the other hand in inhibited GPx4 synthesis, promoting lipid peroxidation. The occurrence of these events causes increased pathology and cellular death. Up and down red arrows indicate the increase and decrease of concentration, respectively.

To date, the etiopathogenesis of ferroptosis in ALS patients is unclear, although there are an increasing number of scientific studies that clearly demonstrate an accumulation of iron in the brain in subjects affected by this pathology [146,147,148]. Obviously, as already mentioned, there are several factors behind ferroptosis, so the correlations between this process and ALS must be numerous, and cannot only refer to iron homeostasis. One of the key players in the development of ferroptosis is certainly the antioxidant system of GPx, which, together with its cofactor GSH, reduces peroxidized lipids and oxidizes GSH to GSSG (see Figure 3). A dysfunction or decrease in GPx activity is implicated in several diseases and neurodegenerations, as widely reported in the literature [149].

Several studies have demonstrated a relation between reduced GPx4 activation in mouse models and neurodegeneration in the cortical and hippocampal areas [19,150]. Wang et al., in a recent paper in 2022, show the key role of this antioxidant system in different transgenic models by demonstrating reduced GPx4 expression in SOD1^G93A^, TDP-43, and C9ORF72 mouse models. Subsequently, to confirm the correlation between ferroptosis and ALS, they obtained a transgenic mouse model in which they overexpressed GPx4, increasing the presence of this antioxidant system in all tissues, including neuronal tissue. Following analyses of SOD1^G93A^ with overexpressed GPx4, a reduced loss of motor neurons and lower levels of lipid peroxidation at the neuronal level were confirmed, validating the cross talk of GPx4 between ferroptosis and ALS [90]. In a similar study, Chen et al. highlighted the overexpression of GPx4 in SOD1^G93A^ mouse models as being correlated with an improvement in ALS. In detail, they compared SOD1^G93A^ and SOD1^G93A^GPx4 mice, the time of onset of the disease, locomotor function (coordination and muscle strength), and motor neuron degeneration, with 4-hydroxynoneal (4-HNE) used as a marker of oxidative stress. For all the parameters considered, it was seen that increasing GPx4 activity improved the neuronal function of mice, thus reducing the onset of ALS [151]. The SOD1^G93A^ mutation is also responsible for the activation of myeloperoxidase (MPO), an enzyme that promotes the formation of hypochlorous acid (HOCl), a powerful oxidant [152]. In neurons, the presence of HOCl increases lipid peroxidation and triggers ferroptosis, which is responsible for neurodegenerative progression [153]. Peng et al. showed that MPO inhibition improved the motor performance of SOD1^G93A^ transgenic mice [154]. Several studies have also shown that a downregulation of Nrf2 occurs in people with ALS, leading to the onset of ferroptosis. The role of Nrf2 in ALS is in line with its known functions in other neurodegenerative diseases. Nrf2 is expressed in various parts of the CNS and has been shown to play a key role in neurodegeneration. Yang et al., in 2023, used the hSOD1^G93A^ mutation in both NSC-34 cells and mice to show Nrf2/GPx4-SLC7A11 axis changes. Indeed, both in vitro and in vivo, the mutation causes a reduced nuclear retention of Nrf2, which leads to the downregulation of SLC7A11 and GPx4 levels, with a subsequent decrease in GSH synthesis and increased oxidative stress. This results in a rapid onset of ferroptosis in models with the hSOD1^G93A^ mutation [155]. From this perspective, Nrf2 can be considered a good pharmacological target in ALS models to decrease the onset of ferroptosis. Sun et al. [153] demonstrated that Nrf2 activator RTA-408 increases the expression of GPx4 and SLC7A11, increasing GSH production, with a significant decrease in oxidative stress alleviating neurodegeneration and symptoms in ALS animal models. So, in conclusion, these findings establish a strong correlation between Nrf2 dysfunction and ALS pathology, particularly in relation to ferroptotic cell death [156]. When Dixon first spoke of ferroptosis, it was defined as a programmed form of cell death, independent of others, caused by an accumulation of iron. However, in autophagy, there is the overexpression of NCOA4 (Nuclear receptor coactivator 4), which causes the degradation of ferritin and dysregulation in free iron homeostasis—hence ferroptosis—so today we often talk about ferritinophagy [157,158]. Although there are no experiments or conclusive data, the fact that NCOA4 is directly implicated in ferritinophagy potentially correlates ferroptosis with ALS, as it is known that neuronal areas such as the putamen, substantia nigra, caudate nucleus, and red nucleus are very susceptible to dysregulated iron levels, and thus to the generation of neurodegenerative diseases like ALS [159,160]. Recently, in 2020, the key role of the enzyme GTP Cyclohydrolase 1 (GCH1) in counteracting ferroptosis was also demonstrated for the first time. This is an enzyme involved in the synthesis of folates and biopterins, which produce BH4/BH2, a potent antioxidant that can counteract lipid peroxidation and ferroptosis-induced cell death [161]. This evidence fits well with the correlation found by Wang et al. between ferroptosis and ALS; they demonstrated that the expression of Speedy/RINGO cell cycle regulator family member A (SPY1) decreases in subjects with ALS due to ubiquitination being mouse double minute 2 homolog (MDM2)-mediated. Subsequently, they showed that in a neuroblastoma hybrid cell line (NSC-34) transfected with hSOD1, an induced overexpression of SPY1 was able to counteract RSL3-induced ferroptosis. The capacity of SPY1 is due to a proportional increase in GCH1 expression (and thus the production of BH4/BH2 and a decrease in TFR1 expression) that causes the import of iron into the cells [162,163]. Also, from a genetic point of view, there is a clear correlation between ferroptosis and ALS; in fact, in 2022, Zhang et al. attempted to confirm this crosstalk using data from the Gene Expression Omnibus, and their analysis revealed some 26 ferroptosis genes that were also altered in subjects with ALS [9,164]. Obviously, of these genes, most are related to the regulation of oxidative stress, and we can consider CYBB (NOX2), unc-51 like autophagy activating kinase 1 (ULK1), hypoxia inducible factor 1 subunit alpha (HIF1A), Transforming Growth Factor Beta Receptor 1 (TGFBR1), and lysosome-associated membrane glycoprotein 2 (LAMP2) to be the most interesting. A very interesting finding is that higher levels of charged multivesicular body protein 5 (CHMP5) are correlated with a shorter life span in ALS sufferers. However, in a later study, CHMP5 is shown to negatively modulate ferroptosis. Dai et al. Fu et al. demonstrated that CHMP5, being part of the endosomal sorting complex required for transport III (ESCRT-III), can reduce lipid peroxidation and ferroptosis-induced membrane damage, and its inhibition leads to an increase in ferroptosis. This apparent contradiction, in which high levels of CHMP5 decrease ferroptosis but also decrease the survival of ALS sufferers, suggests how complex the etiopathogenesis of this neurodegenerative disease is. Certainly, elucidating the role of CHMP5 will require in vitro and in vivo studies in which both pathologies are implicated [165]. Elucidating the relationships between ALS and ferroptosis provides new targets for the treatment of neurodegeneration, because targeting ferroptosis inhibition could emerge as a promising strategy.

## 5. Polyphenols as Potential Ferroptosis Modulators and Therapeutic Agents in ALS

The link between ferroptosis and several neurodegenerative disorders makes it a promising goal for the discovery of new therapies [166]. Specifically, although the exact nature of Ferroptosis-ALS crosstalk is still unclear, to define it represents a formidable challenge for dual therapy research. In this context, natural compounds—particularly polyphenols—are known for numerous activities and exhibit significative properties as ferroptosis modulators [167]. In this paper, a set of polyphenols was evaluated for their therapeutic capacity against ALS and ferroptosis (see Table 1). Polyphenols are widely distributed in plant tissues and are characterized by the presence of multiple phenolic units bound to one or more hydroxyl substituents. Recently it has been demonstrated that some polyphenols are able to cross the BBB and reach the brain, where they often accumulate [168].

### 5.1. Epigallocatechin Gallate (EGCG)

Epigallocatechin gallate (EGCG) is the most abundant and precious component in tea plants (*Camellia sinensis*). It is a water-soluble polyphenolic compound belonging to the catechin class, and has beneficial effects against oxidation, inflammation, cancer, proliferation, and neurological disorders [169]. A study conducted by Koh et al. in transgenic SOD1 mice showed that the antioxidant activity of EGCG in ALS is associated with the upregulation of the Bcl-2 gene [170]. Bcl-2 overexpression has also been shown to play an important role in ferroptosis, inhibiting the level of mitochondrial ROS in duck kidneys [171]. Sarlette et al. have also shown a downregulation of Nrf2 protein levels in the motor cortex and spinal cord of ALS patients compared to controls [172]. Meanwhile, EGCG increased Nrf2 and GPx4 expression, as well as antioxidant capacity in iron-overload mice, demonstrating inhibitory activity towards ferroptosis [173]. Furthermore, several studies highlighted increased skeletal muscle iron accumulation in patients with ALS, and recent studies show EGCG’s ability to inhibit the progression of ferroptosis by acting directly on free iron [174,175]. In detail, Xia et al. demonstrated that EGCG is capable of inhibiting iron overload and lipid peroxidation by inhibiting iron influx and regulating ferritin expression [176]. Iron overload is also one of the triggers that leads to neuroinflammation through the activation of the nuclear transcription factor NF-κB, ROS, and molecular patterns associated with lipopolysaccharide (LPS) present in the outer membrane of Gram-negative bacteria [177]. The inflammatory Fe^2+^/ROS/NF-κB pathway is a crucial link between iron metabolism, cellular inflammation, and ALS; in particular, astroglial and microglial activation in ALS have been associated with LPS-induced inflammation, and LPS injection has been shown to increase the nuclear expression of the CCAAT/enhancer-binding protein δ (C/EBPδ), a transcription factor involved in growth arrest and differentiation whose gene is associated with familial ALS in the spinal cord of G93A-SOD1 mice [178,179]. Experimental studies have indicated the anti-neuroinflammatory effects of EGCG, which is able to enhance LPS-induced microglia activation by inhibiting the TLR4/NF-kB signaling pathway, culminating in the control of inflammatory cytokine gene expression. Furthermore, EGCG also prevented the LPS-induced generation of NO and decreased the expression of cyclooxygenase-2 (COX-2) [180].

### 5.2. Resveratrol (RV)

Another polyphenol with a promising dual activity against ALS and ferroptosis is resveratrol (RV). RV, known as 3,4,5-trihydroxystilbene, is an antifungal molecule of the stilbene family produced in a variety of plant species, notably present in red wine, grapes, and raspberries [181,182]. In addition to the bioactive properties of RV, which include cardioprotective, anti-inflammatory, antioxidant, age-delaying, and antineoplastic effects, several studies have shown that stilbene treatment significantly delays ALS onset [183,184,185,186]. RV significantly extended SOD1^G93A^ mice lifespan and promoted the survival of spinal motoneurons [187,188]. The molecule has shown several beneficial effects that can efficiently counteract ALS and ferroptosis targets; in this regard, Zhao et al. demonstrated the repression of ROS level after RV treatment in ALS mice and that RV, through the overexpression of PGC1*α*, improved motor performance and survival in a mouse model of ALS [189]. In addition, stilbene protects against mitochondrial fragmentation by the activation of PGC1*α*, mediated by RV-SIRT1 interactions [190,191,192]. SIRT1’s natural activation by RV also plays a key role in the regulation of ferroptosis, as it influences the activity of the Xc^−^ system, decreasing the depletion of SLC7A11. In detail, Zhang et al. demonstrated in a heart failure model that, by activating the SIRT1/p53 pathway, RV reduces the degradation of SLC7A11 and increases the levels of GSH and GPx4 in cells [193]. The protective effect of RV on the ferroptosis process is supported by the study of Wang et al., who highlighted that RV reduces lipid peroxidation and ROS production by activating the SIRT3/FoxO3a pathway, balancing the GSH/GPx4 pathway by increasing the expression of SOD2 and catalase [194]. Last but not least, RV ameliorated iron-overload-induced liver fibrosis in mice by regulating iron homeostasis, and in HT22 cells stilbene reduced Fe^2+^ concentrations [195,196]. Iron accumulation has been observed in both sporadic and familial forms of ALS, including in mouse models, and is among the triggers of ferroptosis [197,198].

### 5.3. Kaempferol (KP)

Kaempferol (KP), a flavonoid found in plants and fruits, has potent antioxidant action and a wide range of health benefits, including cardio protection, neuroprotection, antidiabetic properties, and anticancer action [199,200]. Kaempferol protected against the neurotoxicity caused by mutant SOD1 in an ALS model by reducing the intracellular accumulation of the mutant protein and inhibiting the mitochondrial superoxide induced by mutant SOD1. Wang et al. showed that SOD1 amyloid fibril structures caused significant mitochondrial impairment and activated ferroptosis in cell cultures compared to wild-type SOD1 fibrils [130,201,202]. In addition, KP inhibited ferroptosis, reducing the intracellular accumulation of ROS, activating the Nrf2 pathway, and upregulating GPx4 in mouse livers—all common molecular targets in the pathogenesis of ALS [203].

### 5.4. Hesperetin (HST)

Hesperetin (HST), an aglycone derived from hesperidin hydrolysis, belongs to the subclass of flavonoids known as flavanones [204]. It is a compound naturally found in citrus fruits and is widespread in various traditional medicinal herbs using grapefruit peel, orange peel, and tangerine peel. HST exhibits a variety of biological activities, including antioxidant, estrogenic, anti-inflammatory, anticancer, antidiabetic, anti-atherogenic, cardioprotective, and neuroprotective properties [205]. HST exerts its neuroprotective effects through an improvement in endogenous antioxidant defense functions and neural growth factors, and a decrease in neuro-inflammatory and apoptotic pathways. In detail, it attenuates oxidative stress and microglial activation, lowers MDA levels, and elevates CAT, GSH-GPx, and SOD levels. In vitro studies have shown that HST inhibits the generation of ROS, as well as the neuroinflammation associated with the upregulation of Nrf2 and HO-1 [205]. It also enhances neuronal survival through the PI3K-Akt and MAPK pathways and the recruitment of neuronal progenitor cells targeting astrocytes. HST also significantly regulates autophagy through the PI3K/Akt/mTOR/ULK1 pathway, inhibits iron deposition, and reduces damage caused by lipid peroxidation. Additionally, Wang et al. showed that several markers of ferroptosis, such as ACSL4, Gpx4, and ROS generation, were regulated by HST in vivo and in vitro [206,207]. Furthermore, HST can regulate iron-induced cell death by modulating GPx4 and effectively inhibits the elevated levels of intracellular ferroptosis stimulated by erastin by activating SIRT3 [208]. The multiplicity of the molecular targets common to ferroptosis and neurodegenerative diseases, including ALS, makes HST a promising molecule for the development of new drugs and potential treatments.

### 5.5. Quercetin (QC)

Quercetin (QC) is a dietary flavonoid belonging to the subclass of flavonols. The richest source of quercetin is onions; other sources include grapes, cherries, apples, mangoes, citrus fruits, buckwheat, plums, tomatoes, and tea [209]. QC is characterized by the presence of multiple hydroxyl groups that give it powerful antioxidant properties as a free-radical scavenger and chelating agent [210,211]. In particular, its iron-chelating activity inhibits the Fenton/Haber–Weiss reaction and contributes to protection against metal toxicity by promoting iron homeostasis; it also attenuates lipid peroxidation, as well as protein oxidation [194,212]. Taken together, all these properties of QC show promising prospects of treatment for ALS and in general for diseases associated with ferroptosis [213]. In addition, Ruan et al. demonstrated that QC modulates ferroptosis through the activation of the SIRT1/Nrf-2/HO-1 pathway [214]. In addition, Lazo-Gomez et al. demonstrated that QC prevents spinal motor neuron degeneration induced by a chronic excitotoxic stimulus through a SIRT1-mediated mechanism [215]. Thus, QC’s regulation of the SIRT1/NRF2/HO-1 signaling pathway could have effective therapeutic potential against neuroinflammation in neurological diseases [172,216,217]. Moreover, different studies showed QC’s ability to reduce the establishment of toxic SOD1 fibrils, which are clearly involved in the pathogenesis of ALS [218]. In detail, Bathia et al. observed that QC shows moderate binding to native SOD1 but interacts strongly with non-native and higher species of SOD1, suggesting a promising role of QC as an antiamyloidogenic and fibril-destabilizing agent [219]. Wang et al. find a direct link between the amyloid fibrils formed by SOD1 genetic mutations and the GPx4-regulated ferroptosis implicated in ALS [130].

### 5.6. Baicalein

Baicalein is a flavone isolated from the roots of *Scutellaria baicalensis*. It is widely used for its anti-inflammatory, antiviral, and antibacterial properties [220]. Like QC, Baicalein also acts as a potent anti-amyloidogenic and fibril-destabilizing agent for SOD1 fibrils [219]. In addition, in the brain tissues of tMCAO mice, baicalein inhibits ferroptosis processes and ameliorates cerebral I/R injury by decreasing iron levels and lipid peroxidation; in rats, the compound protects cardiomyocytes and I/R-induced ferroptosis via suppressing the accumulation of ROS and malondialdehyde. In pancreatic cancer cells, baicalein inhibits erastin-mediated ferroptosis by counteracting GSH depletion, GPx4 degradation, and lipid peroxidation [221,222]. Thus, in a general context, the compound shows important antioxidant activities effective in the framework of ferroptosis and neurodegeneration. Moreover, in a study conducted by Chang et al., it was seen that the compound is able to restore the TDP-43 aggregates present in oligomeric form in vitro and in diseased cells, confirming the protective action exerted by the compound against ALS [223,224].

### 5.7. Puerarin (PU)

Puerarin (PU), also called daidzein-8-c-glucoside (7,4’-dihydroxy-8-c-glycosylisoflavone), is an isoflavone contained in numerous plants and herbs, including the root of the *kudzu* plant used in traditional Chinese medicine. This molecule possesses multiple biological properties, such as anti-cardiovascular, anti-hyperuricemia, anti-inflammatory, anti-osteoporosis, and anti-ischemia–reperfusion injury properties [225]. Among these beneficial properties, PU shows some common actions towards ALS and ferroptosis, as it modulates Bcl-2 levels, increases antioxidant defenses mitigating oxidative stress, and activates Nrf2/ARE signaling [226]. Chen et al. demonstrated in rat-induced myocardial apoptosis that PU attenuated acute myocardial infarction by upregulating p-PI3K, p-Akt, and Bcl-2 expression and downregulating Bax-cleaved caspase-3 expression [227]. The upregulation of Bcl-2 reduces ROS generation and inhibits ferroptosis, and its downregulation has been shown in the spinal cords of ALS patients and in G93A-mutant SOD1 mice [228]. Alterations in the expression of Nrf2 and Keap1 and the dysregulation of the Nrf2/ARE signaling program have been observed in ALS cellular and animal models and confirmed in human ALS tissue [229,230,231]. Nrf2 plays also an important role in the regulation of ferroptosis, as demonstrated by Chen et al. in lung disease [232]. Furthermore, a study conducted by Wu et al. showed that PU can inhibit iron overload in the cerebral cortex, improving memory and spatial learning disorders in mouse models [233].

### 5.8. 7,8-Dihydroxyflavone (7,8-DHF)

Another flavonoid with potential antioxidant activity against ALS and ferroptosis is 7,8-dihydroxyflavone (7,8-DHF), or tropoflavin. 7,8-DHF is a flavone contained in the leaves of *Godmania aesculifolia*, *Tridax procumbens*, and *primrose* [234]. Several studies show that tropoflavin has therapeutic efficacy against various disorders of the CNS, including neurodegenerative diseases [235]. A study by Korkmaz et al. on transgenic mouse models of ALS (SOD1^G93A^) showed that the administration of 7,8-DHF improves motor performance and lowers motor neuronal survival. In detail, 7,8-DHF, thanks to its ability to bind to the receptor tyrosine kinase TrkB, mimics the functions of BDNF, a neurotrophin that regulates the function, survival, and development of neuronal networks throughout the brain [236,237]. The binding of 7,8-DHF-TrkB leads to the activation of signaling pathways MAPK, PI3/Akt, and ERK1/2 [238,239]. Schiaffino et al. showed reduced total levels of BDNF in SOD1^G93A^ mice in the lumbar spinal cord, and Just-Borràs et al. showed a BDNF/TrkB-FL signaling defect in a mouse model of ALS (SOD1^G93A^ mutation) [240,241]. In addition, chronic treatment with 7,8-DHF for about 3 months improves motor deficits and increases the density of motor neurons in the spinal column [242]. The dysregulation of BDNF levels may induce neurodegenerative mechanisms, such as ferroptosis. Several modulators of the TrkB receptor confer protection against ferroptosis inducers. Specifically, Jakaria et al. demonstrated 7,8-DHF ferroptosis inhibition in immortalized neuronal and non-neuronal cell lines, such as N27 and HT-1080 cells, thanks to its antioxidant properties and ability to trap radicals [243]. 7,8-DHF has also demonstrated anti-inflammatory activity through the suppression of the NF-κB and MAPK signaling pathways, enhancing its beneficial effects on the CNS and its protection against ferroptosis [244].

### 5.9. Fisetin

Fisetin (3,3,4,7-tetrahydroxyflavone) is a flavonol found in a wide variety of plants that exhibits antioxidant, anti-inflammatory, anticarcinogenic, and neuroprotective properties. A study conducted by Wang et al. showed that fisetin improves motor activity, delays the onset of disease, and increases the density of motor neurons in the spinal cord in models of transgenic mice (hSOD1^G93A^), exerting antioxidant and neuroprotective effects in vivo and in vitro; it was observed that there was a decrease in ROS, an increase in the expression of phosphorylated extracellular signal-regulated kinase (ERK), and an upregulation of antioxidant molecules [245]. In addition, fisetin can increase GSH concentration by improving cysteine influx and enhancing glutamate–cysteine ligase activity. Fisetin also has anti-inflammatory effects on microglial cells and inhibits the activity of 5-lipoxygenase by reducing the production of proinflammatory cytokines and lipid peroxidation [246]. A recent study conducted by Yang et al. shows that fisetin inhibits ferroptosis through the activation of the PI3K/AKT/NRF2 pathway and reduces oxidative stress; moreover, the molecule implements cell survival, reduces cell morphological alterations, and inhibits ROS production and lipid peroxidation [247].

**Table 1:** Mechanisms of action of natural compounds in relation to ALS and ferroptosis.

Molecule	Disease	Targets	Functions	References
Resveratrol	ALS	PGC1*α*; SIRT1; iron	Inhibition of ROS; improves the functions of motor neurons	[181,182,184,192]
	Ferroptosis	SIRT1; SIRT3; iron	Promotes Xc^−^ system and SLC7A11; inhibition of ROS; chelates free iron	[188,190]
EGCG	ALS	Bcl-2 gene; iron	Upregulation of Bcl-2 gene; chelates free iron	[164,175]
	Ferroptosis	Nrf2; GPx4; iron	Chelates free iron; inhibition of lipid peroxidation; increases GPv4 activity	[169,171]
Kaempferol	ALS	SOD1 mutant	Reduces aggregation of mutant SOD1	[196]
	Ferroptosis	SIRT1/Nrf-2/HO-1; GPx4	Inhibition of ROS; activation of Nrf2 pathway; upregulation of SLC7A11 and GPx4	[199]
Quercetin	ALS	SIRT1; SOD1 fibrils	Reduces toxic SOD1 fibrils; inhibition of ROS	[214,215,244]
	Ferroptosis	SIRT1/Nrf-2/HO-1; iron	Reduces serum iron by increasing hepcidin expression; inhibition of ROS	[198,210]
Hesperitin	ALS	Nrf2; HO-1; PI3K-Akt pathway; MAPK pathway	Inhibition of ROS; upregulation of Nrf2 and HO-1; regulates ferritin autophagy	[201,202]
	Ferroptosis	PI3K-Akt-mTOR-ULK1 pathway; GPx4; SIRT3	Regulates ferritin autophagy; reduces lipid peroxidation; active GPx4	[203,205]
Baicalein	ALS	TDP-43	Inhibits aggregation of TPD-43	[220]
	Ferroptosis	GPx4; LPO; GSH	Inhibition of ROS; increases [GSH]; increases LPO activity; increases GPx4 activity	[218,219]
Puerarin	ALS and Ferroptosis	Bcl-2; iron; Nrf2/ARE	Inhibition of ROS; reduced [iron]	[221,222,229,245]
7,8-DHF	ALS	Motor neurons; TrkB	Increases motor neuron density	[232,233,234,235,236,237,238]
	Ferroptosis	Radicals	Captures free radicals	[239]
Fisetin	ALS	ERK; GSH; 5-lipoxygenase	Inhibition of ROS; upregulation of antioxidant molecules; increases [GSH]; inhibition of lipid peroxidation	[241,243]
	Ferroptosis	PI3K-AKT-NRF2 pathway	Reducing oxidative stress; inhibition of ROS; inhibition of lipid peroxidation	[243]

## 6. Conclusions

ALS is a disease whose etiology has not yet been fully clarified; there are no effective treatments to reverse its neurodegenerative process, and treatments are mainly symptomatic. However, the relatively recent discovery of ferroptosis as a new form of programmed cell death has opened new avenues towards the search for the molecular processes related to the onset and progression of the disease, allowing the development of potential therapies and new treatments against ALS. In this paper, numerous points of connection between ALS and ferroptosis are highlighted that can no longer be ignored and which, if well focused, can be of great help in the fight against the onset and evolution of neurodegeneration. The main blood markers of ferroptosis, including ferritin, transferrin, and lipid peroxides, are related to the prognosis of ALS. Iron overloads in the brain are a common feature of several neuro-degenerative diseases, including ALS, where iron accumulation is visible in the corticospinal motor pathway before the onset of the disease. Iron species, the key players in ferroptosis, can be harmful and toxic when produced in excess via the Fenton reaction and ROS generation, increasing oxidative stress and inducing neuronal death from ferroptosis. Furthermore, iron overload in glial cells triggers the release of pro-inflammatory cytokines, promoting neurodegeneration. Another altered ferroptosis marker is GPx4 depletion, which has been widely observed in the motor neurons and spinal cords of ALS patients. The delay of symptom onset and the improved survival observed in a SOD1^G93A^ ALS model further support the ferroptosis–ALS relationship hypothesized in this paper. Highlighting this close relationship between the molecular processes leading to ferroptosis evolution and the progression of neuronal degeneration up to the explosion of the disease opens the way not only towards a more detailed knowledge of the molecular mechanisms underlying the disease, but above all allows the discovery of new compounds capable of inhibiting ferroptosis that may have a potential therapeutic use against ALS. In this context, natural compounds with antioxidant properties are undoubtedly a rich source from which to draw. Among these polyphenols, such as those mentioned in our work, seem to be particularly significant because, in addition to their known antioxidant properties, they also act on other molecular targets involved in both ferroptosis and ALS. In this context, the detection of iron at an early stage of the disease, supported by polyphenol anti-ferroptotic activity, might be a highly promising approach for enhancing the effectiveness of conventional therapies. Moreover, polyphenols, as natural substances derived from plants, can be easily obtained, are low in toxicity, and have few side effects and multi-targets. It is of great importance to manage the ALS clinical spectrum with complex biology and significant clinical heterogeneity. Advances in the knowledge of ferroptosis may open the way for the development of new therapies capable of reversing the neurodegenerative process. However, there is a need for further studies to clarify aspects of the extremely complex and interesting, but as yet unclear, relationship between the multifaceted interactions of iron, protein aggregation, and cellular dysfunction that together drive the onset and progression of ALS. Our review has provided information on the relationship between ferroptosis and ALS and suggests the use of polyphenols as potential multitarget agents. This can be used as a basis for further research on new potential therapeutic targets, representing an exciting avenue for treating ALS.

## Data Availability

No new data were created or analyzed in this study. Data sharing is not applicable to this article.
